# A Very Rare Basidiobolomycosis Case Presented with Cecal Perforation and Concomitant Hepatic Involvement in an Elderly Male Patient: A Case Study

**DOI:** 10.3390/ijerph19063412

**Published:** 2022-03-14

**Authors:** Maisa S. Abduh, Saleh M. Aldaqal, Jaudah Almaghrabi, Murad M. Aljiffry, Hany A. Elbadrawy, Majid A. Alsahafi

**Affiliations:** 1 Department of Medical Laboratory Sciences, Faculty of Applied Medical Sciences, King Abdulaziz University, Jeddah 21589, Saudi Arabia; 2 Center of Excellence in Genomic Medicine Research, King Abdulaziz University, Jeddah 22252, Saudi Arabia; 3 Department of Surgery, Faculty of Medicine, King Abdulaziz University, Jeddah 21589, Saudi Arabia; saldaqal@kau.edu.sa (S.M.A.); dr.aljiffry@gmail.com (M.M.A.); hany_kareem1975@yahoo.com (H.A.E.); 4 Department of Pathology, Faculty of Medicine, King Abdulaziz University, Jeddah 21589, Saudi Arabia; jalmaghrabi@hotmail.com; 5 Division of Gastroenterology, Department of Medicine, King Abdulaziz University, Jeddah 21589, Saudi Arabia; majidalsahafi@gmail.com

**Keywords:** Basidiobolomycosis, subcutaneous phycomycosis, subcutaneous zygomycosis, Saudi Arabia

## Abstract

This is a case report of Basidiobolomycosis in a 65-year-old male patient from Jizan presenting with colonic perforation and concomitant liver involvement from February 2021 to July 2021. To control the infection, the patient underwent colonic resection and segmental liver resection, as well as three antifungal drugs. The treatment was successful, and the condition was completely resolved.

## 1. Introduction

Basidiobolomycosis is a rare granulomatous infection of the skin and subcutaneous tissue that can develop quickly and create a persistent infection of the subcutaneous tissue in immunocompetent individuals. It most usually affects infants under the age of one month and is rarely found in adults [1,2].

Even though gastrointestinal involvement is uncommon, it can affect the stomach, small intestine, colon, and liver. Basidiobolomycosis can cause symptoms that are similar to colonic or inflammatory bowel disease. Colonic bowel disease with concurrent liver involvement is uncommon, with only six cases reported. Colonic perforation caused by Basidiobolomycosis is extremely uncommon, with only two cases documented in the literature [2].

In the current case, Basidiobolomycosis was diagnosed in a 65-year-old male who had colonic perforation and liver involvement. A comprehensive literature review was conducted from 1964 to July 2021 to evaluate previously reported cases and gather information on symptoms, diagnostic method, and treatment choices.

## 2. Case

A 65-year-old man from Jizan (southwestern Saudi Arabia) presented with a one-month history of lower abdominal pain, constipation, and weight loss. No significant medical or surgical history was recorded. Ethical approval (HA-02-J-008) was obtained from the Unit of Biomedical Ethics Research Committee at the Faculty of Medicine in King Abdulaziz University, Saudi Arabia.

**Figure 1:**
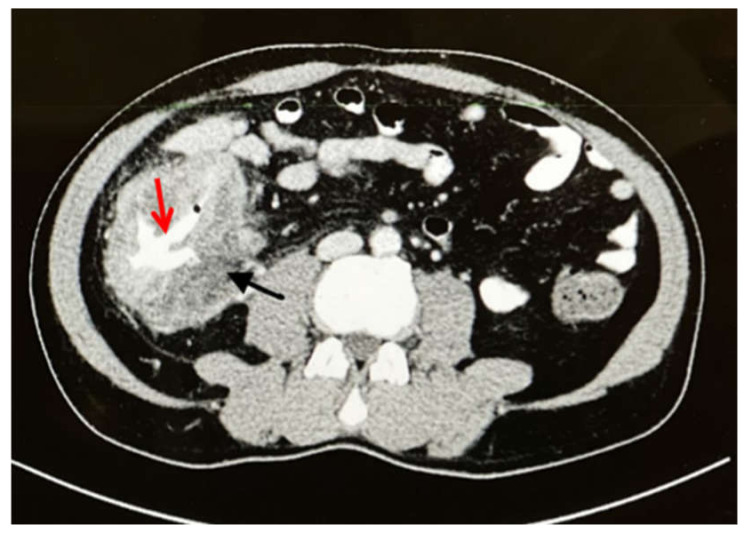
Axial CT with IV and oral contrast showed wall thickening affecting the cecum and ascending colon (**black arrow**) with lumen narrowing (**red arrow**).

Abdominal examination revealed a palpable right-side-abdominal mass with mild tenderness. Laboratory tests revealed leukocytosis (white blood cell (WBC) count: 17,230/mcl), eosinophilia (2540/mcl (14.7%), microcytic hypochromic anemia (hemoglobin (Hb): 10.8 g/dL), and thrombocytosis (platelet count: 505,000/mcl). A computed tomography (CT) of the abdomen revealed circumferential intestinal wall thickening of the cecum and ascending colon, luminal constriction with enlargement of several mesenteric lymph nodes, and two lesions in the liver, segments five and seven, with peripheral enhancement and central hypodensity of 3 × 3 cm (Figure 1).

Colonoscopy revealed an ulcerated, partially obstructed large mass in the proximal ascending colon. A biopsy revealed active eosinophilic colitis with necrotic debris. A liver biopsy of tissue from the mass was performed and showed an eosinophilic abscess. The patient was diagnosed as a case of cecal mass with the possibility of a cancer, colon with liver metastasis need to be rolled out.

**Figure 2:**
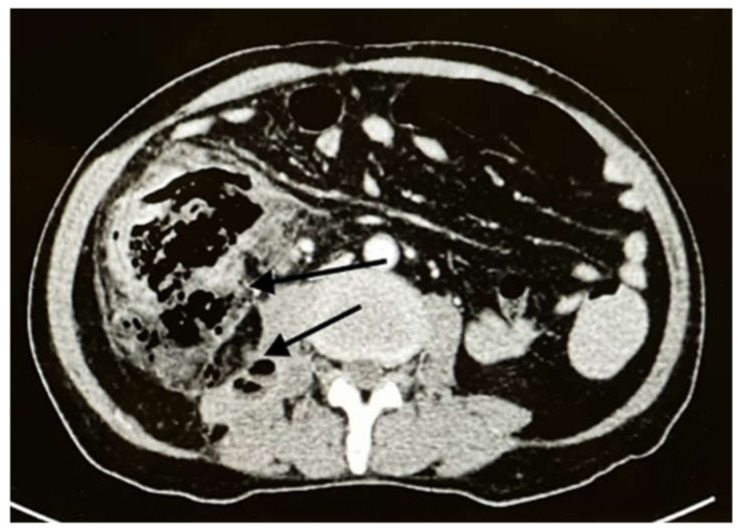
Axial CT with IV contrast showed cecal perforation with free air around the colon (**black arrows**).

**Figure 3:**
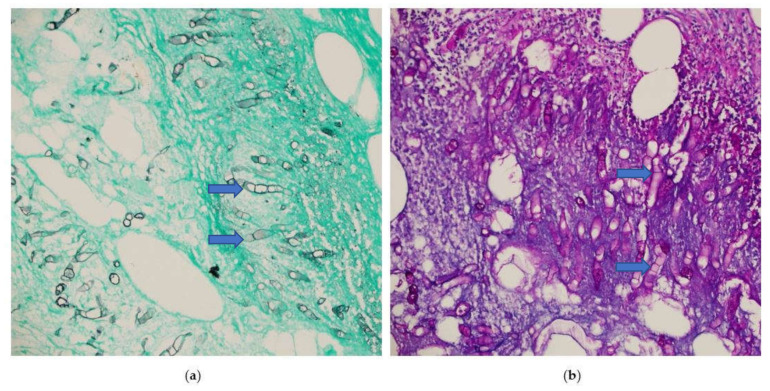
Microscopic sections of the colonic specimen, (**a**) Grocott methenamine silver (blue arrow) (GMS), (**b**) periodic acid-Schiff (PAS) stains shows fungal hyphae that look large, broad, and irregular with sparse septa and thin walls (blue arrow). (Magnification ×400).

The patient returned to the emergency room two weeks later with intense abdominal pain that lasted a few hours. On examination, the patient’s temperature was 38 °C, his pulse was 110 beats/min, and the abdomen was tender with rigidity and an absence of bowel sounds. Laboratory tests revealed the following: WBC count, 13,700/mcl; Hb, 10.4 g/dL; neutrophil count, 9210/mcl (89%); and eosinophil count, 0/mcl (0%). A CT scan of the abdomen showed cecal perforation with free air in the peritoneum and an increase in the size of the liver masses (3 × 4 cm) (Figure 2). Emergency exploratory laparotomy revealed cecal perforation and pus in the peritoneum. Right hemicolectomy with end-to-end anastomosis was performed with drainage of the peritoneum. Intravenous vancomycin (1 g BID for 14 days) was administered to the patient for bacterial peritonitis secondary to colonic perforation. Three days postoperatively, histopathological examination of the resected specimen showed granulomatous inflammation containing multinucleated giant cells associated with eosinophilic infiltrate and micro abscess formation. There were fungal hyphae that look large, broad, and irregular with sparse septa and thin walls. The hyphae were highlighted with special stains that included Grocott methenamine silver (GMS) and periodic acid-Schiff (PAS) stains, the overall features are consistent with Basidobolomycosis infection (Figure 3a,b). Tissue culture from the edge of the perforation in the cecum revealed *Escherichia coli*, *Candida albicans*, and Basidiobolus spp. The patient was started on the antifungal medication voriconazole intravenously (200 mg BID) and was switched to an oral tablet (200 mg BID).

Two weeks later, the patient developed a fever and right-side abdominal pain. Laboratory tests revealed the following: WBC count, 17,230/mcl; neutrophil count, 11,350/mcl (65.9%); eosinophil count 2540/mcl (14.7%); and Hb, 10.8 g/dL. Percutaneous CT-guided drainage was used to drain the abdominal collections. The pleural effusion was aspirated, and percutaneous liver abscess drainage was attempted but failed due to the thick nature of the abscess. Another antifungal medication, itraconazole (200 mg PO. BID), was administered alongside voriconazole (200 mg IV BID) to control sepsis. The patient improved and was discharged from the hospital.

**Figure 4:**
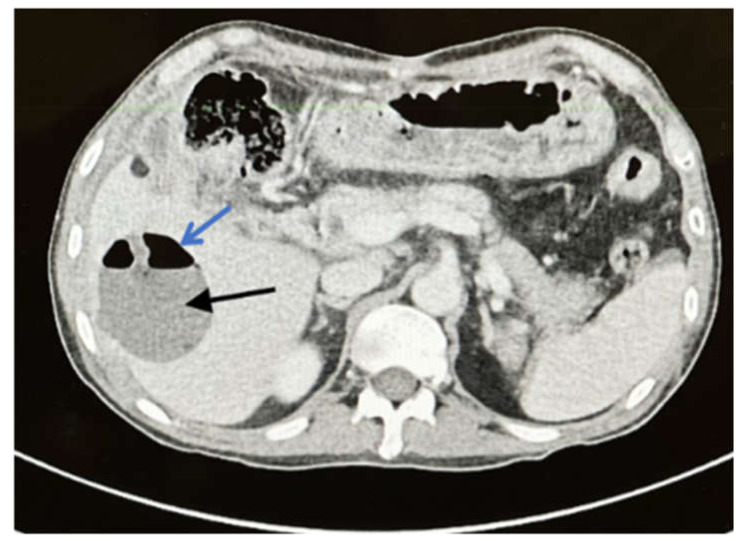
Axial CT scan with delayed contrast phase shows 4 × 3 cm hypo-dense lesion (**black arrow**) with air fluid level (**blue arrow**) in segment six which is characteristic of liver abscess.

Two months later, the patient presented with fever and right upper abdominal pain despite being treated with oral voriconazole (200 mg BID). Upon examination, there was tenderness in the right upper abdomen with low-grade fever. A CT scan of the abdomen showed a liver abscess in segments six and seven (3 × 4 cm and 4 × 2 cm respectively) (Figure 4). Intravenous amphotericin B (225 mg Q24 h) was administered to the patient. Another attempt at percutaneous CT-guided liver drainage failed to drain the abscess. As a result, segments six and seven of the liver were resected segmentally. The patient made a full recovery and was discharged on long-term voriconazole treatment.

## 3. Discussion

Basidiobolomycosis is a rare fungal infection caused by *Basidiobolus ranarum (B. ranarum)*. It belongs to the family Zygomycetes. The Zygomycetes family includes two orders, Mucorales and Entomophthorales. Mucorales cause infection in immunocompromised patients such as diabetics and post-transplants. Entomophthorales, which include Basiodiobolus, cause infection in immunocompetent individuals [3].

Basiodiobolus spp are filamentous fungi that were first identified in 1886 in frogs [4]. They are an environmental saprophyte found in soil and decaying vegetable materials and are occasionally found as commensals in the gastrointestinal tract of amphibians, reptiles, fish, dogs, frogs, and bats.

In 1956, the first case of *B. ranarum* infection in a human (a subcutaneous infection) was reported in Indonesia [1]. Since then, a few cases of subcutaneous, nose, and sinus infections have been reported. Most of the reported cases are from the warmer climates of tropical and subtropical regions, with the majority of reports coming from the southern region of Saudi Arabia, Arizona in the USA, and from Iran [5].

Previous patients were mostly male, with ages ranging from one month to 80 years old [6]. Gastrointestinal Basidiobolomycosis (GIB) is rare. It was first reported in 1964, and there have been 174 cases reported to July 2021. Colonic involvement was reported in 111 cases [7], colonic with liver involvement was reported in six cases [8], and colonic perforation was reported in two cases. The exact route of entry is unknown, but the involvement of the intestine indicates that it is due to the ingestion of food contaminated by infected soil. Table 1 provides a summary of the reported case of Basidiobolomycosis with colonic and liver involvement.

The most common presenting symptoms of GIB are abdominal pain and fever; however, some reported cases presented with colonic obstruction [13], lower GI bleeding, abdominal mass (retroperitoneal or colonic), and colonic perforation (the symptoms in most cases mimicked colonic cancer (abdominal pain, abdominal mass, weight loss), or inflammatory bowel disease (abdominal pain, fever, vomiting, and diarrhea); thus, it is usually misdiagnosed [14].

Leukocytosis, eosinophilia, and anemia were present in most of the cases. Radiological investigation in most of the patients revealed either gastrointestinal wall thickening resembling inflammatory bowel disease, or a colonic mass that mimicked colonic cancer, with or without a concomitant liver mass.

Endoscopy and biopsy are inconclusive in nearly all reported cases. Therefore, a mucosal biopsy is usually performed, and *B. ranarum* grows in the submucosa, so the histological examination of the biopsy commonly reveals inflammatory changes with eosinophilic infiltration and, in some cases, the presence of granulomas.

All reported cases were diagnosed either by excisional biopsy of tissue from the mass or histological examination of the resected segment of the colon, which is considered the gold standard for diagnosis. Histological diagnosis usually reveals the diagnostic features of this fungal infection, namely the presence of fungal hyphae with Splendore–Hoeppli bodies, granuloma with abundant eosinophilia, and intensely radiating eosinophilic granular material. A few reported cases were diagnosed by polymerase chain reaction (PCR) and molecular methods such as DNA sequencing of samples obtained from equivocal tissue specimens.

Treatment with antifungal therapy without surgical resection of the affected part has been successfully reported in a few cases [15]. In these cases, antifungal therapy was administered for an extended period. In most cases, antifungal therapy was administered for 6 months or more. Mortality from GIB is rare and usually in pediatric patients, except for two reported cases of a 61-year-old and 18-year-old with disseminated disease, they died despite antifungal therapy and surgery [7,9]. Awareness of the disease amongst physicians and pathologists would contribute to the successful treatment of more recent cases and would result in a significant reduction in the disease’s morbidity and mortality rates. Further research on the risk factors that lead to GIB are highly recommended.

## 4. Conclusions

GIB can be associated with a variety of signs and symptoms, and it can affect people of all ages. Most GIB cases presented with colonic involvement, few with liver involvement, and very rare cases presented with colonic perforation and concomitant liver involvement, as in this study. The diagnosis of GIB requires a high index of suspicion, and we recommend its inclusion in the differential diagnosis of abdominal masses associated with fever, weight loss, and eosinophilia, especially in endemic regions.

## Figures and Tables

**Table 1:** Summary of the reported cases of Basidiobolomycosis with colonic and liver diseases.

Patient Information & Disease	Antecedents	Hospitalization Cause?	Type of Infection	Treatment	Follow-Up Patient	References
61-year-old man (Division of Infectious Diseases, Tropical Medicine and AIDS unit in Amsterdam Hospital (Netherlands)).	Progressive left abdominal pain and constipation for a few months. Colonoscopy showed an obstructing tumor in the descending colon, and a hemicolectomy was performed. Histology showed inflammation, possibly caused by a fungal or parasitic infection, without definite identification of an organism. A few weeks postoperatively a CT scan, made because of abdominal discomfort, revealed a liver mass (6 cm). Treatment with metronidazole, directed against an amoebic liver abscess was unsuccessful.	Gastrointestinal Basidiobolomycosis with an obstructing colon tumor and a large hepatic mass.	A presumptive diagnosis of *Basidiobolus spp*. infection after autopsy *Basidiobolus ranarum* was cultured from liver, gallbladder and colon	Treated with amphotericin B (itraconazole contraindicated because of renal insufficiency).	A few days later the patient died of septic shock.	[9]
12-year-old boy (Yemeni boy living in Abha, Aseer, Saudi Arabia).	2-month history of diffuse abdominal pain, non-bilious vomiting, poor appetite, and weight loss.	The initial provisional diagnosis was intestinal lymphoma, and a right hemicolectomy was carried out, but histopathological assessment ruled out lymphoma and suggested intestinal tuberculosis. Two weeks after starting antituberculosis medications, the patient was referred to our hospital because of fever and right upper abdominal discomfort. There was leukocytosis with marked eosinophilia, and a liver biopsy showed evidence *of B. ranarum* infection.	Gastrointestinal Basidiobolomycosis with hepatic dissemination.	Itraconazole treatment was started immediately at a dose of 100 mg twice daily.	The patient was healthy.	[10]
41-year-old woman (Shiraz, Iran).	She was complaining of abdominal pain, nausea, and experienced significant weight loss for one month. Past medical history and personal history were not significant, and she had no specific risk factor exposure.	Suffered from Basidiobolomycosis with concomitant lesions in the cecum and liver involvement. Physical examination temperature was 38.5 °C (101.3 °F) and signs of anemia, including pale conjunctiva were obvious. Also, she had mild generalized abdominal tenderness with no peritoneal sign.	Gastrointestinal Basidiobolomycosis infection with concomitant lesions in the cecum and liver involvement.	Patient was treated with itraconazole 200 mg twice a day for 4 months. The patient health status showed significant improvement, and no evidence of active liver lesion was detected in follow-up imaging study.	In the next year of follow-up, the patient was healthy and symptom free.	[11]
5-year-old boy (Bushehr, Iran).	2-month history of diffuse abdominal pain, non-bilious vomiting, poor appetite, weight loss, and a detectable mass on abdominal sonography.	This study presents a boy with colonic BM involving the liver, masquerading as gastrointestinal lymphoma. Physical examination, he seemed ill and emaciated, his body temperature was 39 °C, and his liver was tender on palpation 3 cm inferior to the costal margin. Multiple lymphadenopathies were detected. The mass was similar to Castleman’s disease, lymphoma, or tuberculosis.	Colonic BM involving the liver, masquerading as gastrointestinal lymphoma.	Posaconazole as an effective single agent treatment with minimum complications during a prolonged treatment plan. Treatment with amphotericin B (intravenous 1 mg/kg/day for 2 months) and Posaconazole (200 mg by mouth four times per day).	The patient was followed closely by means of physical exams and abdominal computed tomography scans, which revealed no recurrence 6 months after starting therapy with Posaconazole.	[12]
18-year-old women (Jazan, Saudi Arabia).	2-week history of right lower quadrant abdominal pain associated with nausea, vomiting and anorexia. Medical and surgical history were unremarkable, and she was not on any medications.	Obstructing cecal mass initially suspected to be malignant.	Surgical resection was complicated by bowel perforation, histology and cultures confirmed Basidiobolomycosis infection.	The postoperative course was complicated by an enterocutaneous fistula, fungal intraabdominal abscesses, liver and lung abscesses, formation of mycotic hepatic artery aneurysm and meningoencephalitis. This was treated successfully with vacuum-assisted closure device and total parenteral nutrition (TPN).	Despite aggressive intensive care unit and antimicrobial treatment, she expired due to septic shock.	[7]
39-year-old woman (Southern part of Saudi Arabia).	Hypertension, for which she was being treated with amlodipine. She had visited several hospitals and had provisionally been diagnosed as having either a retroperitoneal malignancy or retroperitoneal fibrosis before being referred to hospital.	Severe left-sided abdominal pain and weight loss.	Retroperitoneal Basidiobolomycosis infection.	Antifungal treatment. This led to significant improvement, without surgical intervention.	Currently, she is still on treatment and undergoing follow-up.	[2]
